# Awareness of Preeclampsia among Antenatal Clinic Attendees in Northwestern Nigeria

**DOI:** 10.1055/s-0043-1770700

**Published:** 2023-07-03

**Authors:** Aisha N. Adamu, Katie L. Callahan, Peter B. Anderson

**Affiliations:** 1 Department of Obstetrics and Gynecology, Federal Medical Centre Birnin Kebbi, Kebbi State, Nigeria; 2 Department of Community Health Education and Recreation, University of Maine at Farmington, Farmington, Maine, United States; 3 Contributing Faculty and College of Health Professions, Walden University, Minneapolis, Minnesota, United States

**Keywords:** preeclampsia, antenatal clinic, maternal mortality, awareness, Nigeria, low- and middle-income countries

## Abstract

**Background** Preeclampsia (PE) is among the five main causes of maternal mortality in low resource countries. This study was designed to assess PE awareness and its socioeconomic determinants among antenatal clinic attendees in northwestern Nigeria.

**Methods** Two hundred twenty-one antenatal clinic attendees in northwestern Nigeria were selected through systematic random sampling for this quantitative study. Women who were 9 months pregnant and had consented to participate were included; those with chronic illnesses such as diabetes mellitus were excluded. Data on respondents' sociodemographic variables, and PE awareness were collected using a validated questionnaire. Associations between variables were tested using chi-square test and multiple regression analysis.

**Results** Ninety-one percent of respondents were aged 20 to 40 years, 53.9% were multiparous, 27% had no or low level of formal education, and 52% had attended antenatal care (ANC) at least four times in the index pregnancy. Only 37% ( *N* = 83) were aware of PE. Women with formal education were 3.8 times more likely (odds ratio [OR] = 3.8, 95% confidence interval [CI] = 1.4–10.3) to be aware of PE compared with those with no formal education ( *p* < 0.05). Also, women who experienced hypertension in their previous pregnancies were 2.8 times more likely (OR = 2.8, 95% CI = 1.37–5.71) to be aware of PE than those women who had not ( *p* < 0.05).

**Conclusion** There was a low level of PE awareness among pregnant women in this study; being formally educated and having had hypertension in a previous pregnancy were positively associated with PE awareness. PE education should be part of ANC.

## Introduction

Maternal and perinatal morbidity and mortality rates are high among low- and middle-income countries (LMICs). The World Health Organization reported a maternal mortality ratio of 239 deaths per 100,000 live births compared with just 12 per 100,000 live births in developed countries in 2015. 1 Maternal mortality figures in Nigeria are estimated to be approximately 800 deaths for every 100,000 live births, with the highest figures recorded in the northern regions of the country. 2 Preeclampsia (PE) is an important cause of maternal and perinatal morbidity and mortality, especially in LMIC, and a major contributor to the high morbidity and mortality figures among women and children. 3 PE is one of the five major causes of maternal and perinatal morbidity and mortality worldwide. 4 5 Globally, up to 76,000 women die from the disease annually, and 99% of these deaths occur in developing countries where resources are limited. 1 6 In Nigeria, a PE-related maternal mortality rate of 1.9% has been reported. 7 These mortality figures could be reduced by early disease detection and appropriate management during the antenatal period as this could improve the clinical outcome for both the mother and baby. 1 8 Antenatal care (ANC) attendance permits the early identification of women with elevated blood pressure (BP) that is PE, and also permits close monitoring of maternal BP in those who have developed it 9 to prevent or reduce the risk of PE-related maternal and perinatal outcome. 10 PE awareness and knowledge among pregnant women improves their responsiveness to the symptoms of PE, and empowers them to make appropriate decision (such as ANC attendance) about their health. 11 12 Awareness about the disease and its complication could positively influence early ANC booking and compliance with subsequent antenatal visits.

Worldwide, there is a low level of awareness about PE and its severity, 11 but strategies to control PE have focused more on increasing the knowledge and skill base of care providers and boosting the capacity of health facilities to provide appropriate PE care. 13 However, it may not only be the knowledge of care providers that needs to be improved upon. Previous studies suggest that lack of knowledge among pregnant women themselves regarding PE also needs further attention and research. Mosca et al 14 studied knowledge about PE in a heterogeneous online community of women aged 20 to 35 years. They found a low level of knowledge about PE among the study population and recommended focusing on women when disseminating information about PE in the community.

The purpose of this quantitative study was to explore pregnant women's awareness about PE in a low resource setting where poor community understanding of the disease could delay seeking appropriate care. 15 The information obtained could guide the design and implementation of public health campaigns targeted at reducing PE-related maternal morbidity and mortality.

## Methods

This quantitative cross-sectional study used three sites that were selected at random from a population of health facilities in the seven states of the northwestern region of Nigeria. These were facilities concerned with the provision of ANC services and that also had the capacity to manage women who developed PE in the course of their pregnancies. From these three sites a total of 221 women were surveyed. The sample size was determined using the G*Power software 16 assuming that 50% of pregnant women in the population were aware of PE and that any significant relationship between PE and its predictors would be proven at a *p* -value of 0.05 ( *α* -level = 0.05) and a power of 0.8 if such a relationship existed. The number of participants per facility was determined proportionately based on the total antenatal clinic attendance at the facility in the preceding year and these latter were 3,350, 3,850, and 13,508 for each of the three facilities. From these, 43, 50, and 128 respondents were sampled consecutively from the three facilities, respectively, using a sampling frame of 20,218, a sample size of approximately 221, and the sampling fraction of 0.01.

The study population were women who booked for and received care at the facilities, were at least 9 months pregnant, and had consented to participate in the study. Women who had any form of chronic illness (such as systemic hypertension, diabetes mellitus, sickle cell disease, or asthma), were not included because it was assumed that this category of women would, by the nature of their illness, visit the hospital more often and so would have the possibility of potentially higher chances of access to health information than the others. The purpose of the research was explained to the women who had met the inclusion criteria; this was done after their antenatal health talks. Thereafter, every pregnant woman who showed interest in participating was approached individually and privately for consent administration.

The data collection instrument was a structured questionnaire developed from a comprehensive review of relevant literature and data collection tools used by other researchers in similar studies. 17 It consisted of structured questions that assessed the demographic and clinical variables of the respondents (the covariates) including questions to ascertain the number of antenatal visits achieved. The demographic and clinical variables included respondents' age, marital status, parity, level of education, and maternal income (i.e., the covariates). To assess their PE awareness, respondents were given a brief description of what the condition was and they were then asked if they had ever heard of it, their responses were documented as “yes” or “no.” Through a multiple response question, respondents were also asked if they had experienced PE/E and/or any of a list of other complications in their previous pregnancies.

Once developed, the questionnaire was validated in five main steps of (1) establishing face, content, and construct validity, and consensual validation, (2) running a pilot test, (3) cleaning the data collected, (4) checking for internal consistency using Cronbach's *α* , and (5) revising the questionnaire. Face and content validity was provided by specialists relevant to the field of study. 18 The data generated were analyzed using the Statistical Package for Social Scientist version 27 and results were presented in frequencies and percentages. Association between respondents' PE awareness and their sociodemographic and obstetric characteristics were tested using chi-square test and multiple logistic regression.

Ethical approval for this research was first obtained from the Research Ethics Committees of each of the selected health facilities, after which a final approval was obtained from the Institutional Review Board of Walden University, United States, as the supervising institution for the research (approval number 12–23–20–0417876).

## Results

About 27% of the study population had no or low level of formal education. Almost all the respondents were married at the time of this study, 91% of them were between 20 and 40 years of age, only 16.7% were pregnant for the first time, and the others were either multiparous 53.9%, or grand multiparous 29.4%. In terms of education, 14.5% had no formal education, while the rest had a secondary level of education (49.3%, *N* = 109). About 28% of them booked at 28 weeks of gestation or more, only 4.5% booked before the 16th week while the rest booked for ANC at a gestational age of between 16 and 27 weeks. Fifty-two percent of the population had attended ANC at least four times during the course of the index pregnancy. Of those who had been pregnant before, 50.5% ( *N* = 93) had experienced a complication and 20.7% ( *N* = 38) had had elevated BP in a previous pregnancy. Only 37% ( *N* = 83) of the respondents had ever heard of PE. The chi-square distribution of the respondents' characteristics by their PE awareness was also calculated; having some formal education (chi-square = 7.67, degrees of freedom [df] = 2, *p* = 0.01) and having had elevated BP in their previous pregnancy (chi-square = 6.22, df = 1, *p* = 0.01) were the two respondent characteristics that were significantly associated with awareness about PE. Details of these analyses are shown in Table 1 .

**Table 1:** Descriptive statistics of sociodemographic and obstetric characteristics of the respondents

Predictive variables	Number of respondents	Not aware *N* = 138 (62.4%)	Aware, *N* = 83 (37.6%)	Chi-square	*p* -Value
Age range (in years) < 20 20–30 31–40 > 40	12 136 65 8	8 (5.8) 83 (60.1) 39 (28.3) 8 (5.8)	4 (4.8) 53 (63.9) 26 (31.3) 0 (0)	5.18	0.16
Parity Primigravida Multipara Grand multipara	37 119 65	28 (20.3) 68 (49.3) 42 (30.4)	9 (10.8) 51 (61.5) 23 (27.7)	4.32	0.12
Respondent has formal education No Yes	32 189	27 (19.6) 111 (80.4)	5 (6.0) 78 (94)	7.67	0.01
Respondent has a source of income Yes No	142 79	82 (59.4) 56 (40.6)	60 (72.3) 23 (27.7)	3.74	0.06
Monthly income category a ⩽ N36,000 > N36,000	130 12	74 (53.6) 8 (5.8)	56 (67.5) 4 (4.8)	0.43	0.51
GA at time of antenatal registration < 16 wk 16–21 wk 22–27 wk ⩾ 28 wk	10 95 68 48	6 (4.3) 58 (68.8) 41 (29.7) 33 (23.9)	4 (4.8) 37 (44.6) 27 (32.5) 15 (18.1)	1.06	0.78
Number of ANC visits < 4 visits ⩾ 4 visits	106 115	67 (48.6) 71 (51.4)	39 (47.0) 44 (53.0)	0.05	0.82
Had HTN in previous pregnancy b No Yes	146 38	94 (85.5) 16 (14.5)	52 (70.3) 22 (29.7)	6.22	0.01

Abbreviations: ANC, antenatal care; GA, gestational age; HTN, hypertension.

a *N* = 142, income in Naira at rate of N36,000 to 1 US dollar at the time of data collection.

b *N* = 184.

The nature of the relationship between PE awareness and those variables with which it was significantly associated (see Table 1 ) was explored further using multiple logistic regression analyses; the odds of women with formal education to be aware of PE was 3.8 (odds ratio [OR] = 3.8, 95% confidence interval [CI] = 1.4–10.3; *p* < 0.05), while the odds of women with previous hypertension to be aware of PE was 2.8 times more likely (OR = 2.8, 95% CI = 1.37–5.71; *p* < 0.05). The detail of this result is shown in Table 2 .

**Table 2:** Multiple logistic regression analysis of respondent characteristics by PE awareness

Predictor	*B*	SE	Wald chi-square	df	Significance	Odds ratio (95% CI)
Respondents with formal education No Yes	1.196	0.57	4.46	1	0.04	1.00 a 3.31 (1.089–10.04)
Respondents who had hypertension in a previous pregnancy No Yes	1.070	0.42	6.61	1	0.01	1.00 a 2.91 (1.21–4.21)
Parity of respondents by category Primigravida Multipara Grand multipara	−0.78 −0.03	0.64 0.43	1.45 0.01	1 1	0.23 0.94	1.00 a 0.47 (0.13–1.62) 0.97 (0.41–2.27)
Do you have a source of income? No Yes	0.50	0.32	2.38	1	0.12	1.00 a 1.65 (0.87–3.12)
Age group of respondents ≤ 19 20–29 30–39 ≥ 40	−0.27 −0.35 −21.30	0.74 0.84 13228.32	0.14 0.18 0.00	1 1 1	0.71 0.68 0.99	1.00 a 0.76 (0.18–3.26) 0.70 (0.14–3.62) 0.00 (00)

Abbreviations: CI, confidence interval; df, degrees of freedom; PE, preeclampsia; SE, standard error.

Note: *R* ^2^ = 0.13 (Cox and Snell), 0.17 (Nagelkerke).

a Reference category.

## Discussion

The majority of the respondents fell within the age bracket of 20 to 40 years with only a small percentage falling at the extremes of the reproductive age. The proportion of teenage mothers in this study was lower than the 21% reported for the region in the Nigeria National Demographic and Health Survey (NDHS). 19 It is difficult to provide a precise explanation for why this was so, but it may be related to the nature of the antenatal clinic attendance schedule in one of the study sites and the possibility of undersampling. In one of the study sites, antenatal visit days were scheduled based on parity; all primigravida attended on the same day of the week, all multipara on the same day, and all grand multipara on the same day. Since teenagers were more likely to belong to the group of primigravida, it is possible to have had an underrepresentation of this category depending on the number of women who attended on each of their antenatal visit day.

The respondents in this study were of high parity, as much as one-third of them have had at least four previous deliveries and only 16% of them were pregnant for the first time. While grand multiparity may not be a significant risk factor for the development of PE, it is an important risk factor for other causes of maternal mortality such as postpartum hemorrhage and ruptured uterus 20 ; the northwestern region has been reported to have high maternal mortality rates. 19 Although the focus of our research was not on parity, the finding of high parity in this research could serve as an advocacy point for other maternal health-related public health activities such as family planning. In the NDHS report of 2019 the contraceptive prevalence rate among currently married women in the surveyed population of the northwestern region of Nigeria was just 6.8%. 19

The proportion of women who were aware of PE in this research was low (37.6%), even though up to 52% of the respondents visited the antenatal clinic four times or more. This finding may not be significantly different from what has been reported in other places where PE is also prevalent, 10 11 21 22 but it puts to question the depth of information and the quality of the health talks provided to women at their ANC visits. In a study by Hutchinson, 22 women were reported to be poorly educated about PE at ANC, a finding the researcher felt could be a significant contributor to delays in seeking care for PE affected women. Even where awareness about the possible complications associated with the condition is reported to be good, myths still existed regarding the risk factors associated with its actual occurrence. 23

Factors that were significantly associated with awareness of PE included educational level of the mothers as well as their experience of elevated BP in a previous pregnancy. Women were more likely to be aware of PE if they had formal education compared with those without, this was so for all women regardless of their parity. The relationship demonstrated between formal education and PE awareness in our study was not unexpected because it is known that education is associated with better ability to access health information. 24 This relationship is somewhat different from that between educational level and eclampsia where some researchers have reported a nonsignificant association between education and eclampsia awareness. 25 One may be quick to note that, unlike PE, eclampsia is very dramatic in its occurrence and it usually draws attention when it occurs thereby making it easy to recognize and remember; so PE may exist unnoticed by the pregnant woman or her relations. 26 In such a situation, awareness about the existence of PE may be influenced by how much a person knows of, and can recognize its features in women who have it. We did not set out to assess how respondents became aware of PE in our study, but it is known that women who are formally educated are more likely to access information about health issues from sources other than antenatal clinics. 27 In their study on assessing knowledge of PE/E among women in Ghana. 28 Fondjo et al reported a significant association between respondents' educational level and knowledge of the condition. 28

For women who had delivered before and those who had experienced a hypertensive disease in their previous pregnancies were significantly more likely to be aware of PE compared with those who had not. Perhaps their previous experience of hypertension exposed them to information about hypertension which they now related to PE which is also a form of hypertensive disease. Previous studies have shown that experiencing one health condition could positively impact awareness of another related health condition as demonstrated by Mirzaei et al in their study where having diabetes mellitus was found to be a significant predictor of awareness about hypertension. 29 Also, since the respondents in our study were not asked to state the type of hypertensive disorder they had in the previous pregnancy, it is possible that they may have actually had PE which may then have raised their awareness about the condition. Brown et al 30 studied women who had experienced PE in their previous pregnancies, and they reported that such women were more aware of the likelihood of recurrence of the condition in a subsequent pregnancy. Previous experience of a disorder could therefore raise awareness about that disorder.

The finding of low level of awareness as reported by the respondents in our study may also be related to their level of recall; depending on the importance they attach to it, pregnant women may not remember all the information they are provided with in the antenatal period. 31 Other researchers have also found poor health care provider communication skills as contributory to low level of maternal health information among antenatal clinic attendees. 27

## Study Limitations

The study was conducted through a rigorous application of the chosen methodology, however, limitations still exist. First, there is the possibility of selection bias and this has been mentioned in the text; the structure of the antenatal clinic schedule at one of the study sites may have led to over- or undersampling of a parity group. In addition, the sample size may have been limited by the amount of time available to the researchers to conduct the study, it is possible that with a larger sample size the nature of the association between PE awareness and other variables may be different.

## Conclusion and Recommendation

Awareness about PE was poor among antenatal clinic attendees in northwestern Nigeria and this was so regardless of the number of antenatal clinic visits achieved by women in this study. Awareness was, however, found to vary with the respondent's level of education and their previous experience of hypertension in pregnancy. PE is an important cause of maternal morbidity and mortality, so this finding of low level of awareness about the condition should be a cause for concern especially to public health professionals who are interested in making pregnancy outcome better for women. The finding should galvanize action for improved antenatal health education as well as public campaigns to improve PE awareness among the general public. Women with no formal education should be specifically targeted with specialized programs and strategies to provide them with maternal health information, including PE as they have been demonstrated to have a lower likelihood for PE awareness in this study.
